# A Comparison of Endoscopic Ultrasound-Guided Fine-Needle Aspiration and Fine-Needle Biopsy in the Diagnosis of Solid Pancreatic Lesions

**DOI:** 10.1155/2018/1415062

**Published:** 2018-04-19

**Authors:** Lachlan R. Ayres, Elizabeth K. Kmiotek, Eric Lam, Jennifer J. Telford

**Affiliations:** ^1^Poole Hospital NHS Foundation Trust, Poole, UK; ^2^Jagiellonian University Medical College, Krakow, Poland; ^3^Division of Gastroenterology, St. Paul's Hospital, University of British Columbia, Vancouver, BC, Canada

## Abstract

**Background and Aims:**

Endoscopic ultrasound (EUS) guided fine-needle aspiration (FNA) is the method of choice for sampling pancreatic lesions. This study compares the diagnostic accuracy and safety of FNB using a novel core needle to FNA in solid pancreatic lesions.

**Methods:**

A retrospective review of patients in whom EUS FNA or FNB was performed for solid pancreatic lesions was conducted. Diagnostic performance was calculated based upon a dual classification system: classification 1, only malignant pathology considered a true positive, versus classification 2, atypical, suspicious, and malignant pathology considered a true positive.

**Results:**

43 patients underwent FNB compared with 51 FNA. Using classification 1, sensitivity was 74.0% versus 80.0%, specificity 100% versus 100%, and diagnostic accuracy 77.0% versus 80.0% for FNB versus FNA, respectively (all *p* > 0.05). Using classification 2, sensitivity was 97% versus 94.0%, specificity 100% versus 100%, and diagnostic accuracy 98.0% versus 94.0% for FNB versus FNA, respectively (all *p* > 0.05). FNB required significantly fewer needle passes (median = 2) compared to FNA (median = 3; *p* < 0.001). Adverse events occurred in two (4.5%) FNB patients compared with none in the FNA group (*p* > 0.05).

**Conclusion:**

FNA and FNB have comparable sensitivity and diagnostic accuracy. FNB required fewer passes.

## 1. Introduction

Endoscopic ultrasound (EUS) guided fine-needle aspiration (FNA) is the method of choice for evaluating and sampling solid pancreatic lesions [1–3]. EUS can detect cancers less than 10 mm in size and is more sensitive than computed tomography (CT) [4]. It has a safe, cost-effective, and highly accurate method to diagnose solid pancreatic mass lesions [2, 4–7].

There is uncertainty relating to the optimal needle gauge, number of needle passes, presence of an on-site pathologist, and more recently whether the ability to procure core samples using fine-needle biopsy (FNB) is advantageous [3, 8–11]. Several core biopsy needles are available which have the potential to preserve tissue architecture and morphology which is helpful for the characterization of some lesions such as stromal tumors and lymphomas [12–14]. Generally, FNB is comparable to FNA in terms of diagnostic accuracy for solid pancreatic lesions [13–17].

The recently developed SharkCore™ FNB needle is designed with six cutting edge surfaces and an opposing bevel to trap core tissue which preserves architecture and limits tissue fracturing in addition to including a passively activated safety sheath to prevent needle stick injuries. A recently published pilot study demonstrated comparable diagnostic performance compared to FNA [18].

The aim of this study was to compare the sensitivity, specificity, and safety of SharkCore FNB to conventional FNA in evaluating solid pancreatic masses.

## 2. Methods

A retrospective review was performed on consecutive patients who underwent index EUS guided FNB of solid pancreatic lesions by two experienced endosonographers at St. Paul's Hospital, Vancouver, BC, using the Covidien SharkCore platform (Shark Core®, Covidien, Dublin, Leinster, Ireland) using 19 G, 22 G, or 25 G needles. When the study was conceived 50 FNB had been performed on solid pancreatic lesions (November 2014 to July 2015). Thus a similar number of consecutive patients undergoing FNA using a 22 G or 25 G needle (Expect, Boston Scientific, Natick, MA, USA) of solid pancreatic lesions were taken for comparison (from October 2013 to October 2014). There was no on-site pathologist present for either cohort. The number of needle passes and needle throws was not standardized and was at the discretion of the endosonographer. Assessment of an adequate specimen was also at the discretion of the endosonographer generally using a crude visual assessment of the material expressed from the needle.

Patients were excluded when there was a predominantly cystic component to the mass or if adequate follow-up was not available: either surgical pathology or six months' clinical follow-up. The study was approved by the University of British Columbia Ethics Board.

Electronic medical records were interrogated and demographic data recorded. The size of the lesion was documented based on the largest dimension reported in millimeters. The location of the lesion was categorized as falling within the head, uncinate, genu, body, or tail of pancreas. Technical failures, number of needle passes, and needle gauge were recorded.

The pathological diagnosis by EUS was categorized as nondiagnostic, benign, atypical, suspicious, or malignant (i.e., classes 1–5, resp.). Diagnostic accuracy, positive predictive value, negative predictive value, sensitivity, and specificity were calculated using a dual classification system employed in a meta-analysis of EUS FNA by Hewitt et al. 2012 [2]. Under this approach malignancy status was established as follows:

- Classification 1: nondiagnostic, benign, atypical, and suspicious are considered negative for malignancy. Only the designation “malignant,” that is, class 5, is considered a true positive.
- Classification 2: nondiagnostic and benign are negative for malignancy. Atypical and suspicious are also considered positive for malignancy.

Diagnostic accuracy was compared to gold standard surgical pathology, subsequent EUS FNA/FNB, or six-month clinicoradiological follow-up. It was computed as the ratio between the sum of true positive and true negative values divided by the total number of lesions. Neuroendocrine tumors which have a range of malignant potential were all regarded as a “positive” diagnosis and grouped with adenocarcinoma and lymphoma for the purposes of the analysis. Adverse events, as determined by interrogating electronic medical records, were also recorded and compared.

### 2.1. Statistical Analysis

Statistical analysis was performed using the chi-square test, Fisher's exact test, or Wilcoxon rank sum test as appropriate using SAS 9.4 and R 3.2.0. A *p* value of ≤0.05 was considered statistically significant.

## 3. Results

### 3.1. Patient Demographics

Patients ranged from 33 to 88 years of age (median = 66 years of age for both groups). There were 22 (43.1%) versus 25 (58.1%) males in the FNA and FNB groups, respectively. Demographic data of the study population is reported in Table 1.

### 3.2. Lesion Characteristics

There was no difference in location, size, or pathological class (all *p* > 0.05) between the two groups. Lesion characteristics are shown in Table 2. Most lesions were located in the head of pancreas (61.7%) followed by the body (13.8%), tail (10.6%), uncinate (7.4%), and genu (6.4%). The mean lesion size was 27 mm (± 12.2 (SD)), 72.3% were malignant, 10.6% suspicious, 6.4% atypical, 8.5% benign, and 2.1% nondiagnostic. The majority of lesions (68%) were pancreatic ductal adenocarcinoma with neuroendocrine lesions the second most common. The two groups differed significantly in terms of the final diagnosis (*p* = 0.018) in that there were more neuroendocrine tumors (NET) in the FNA group and more inflammatory lesions and lymphomas in the FNB group.

### 3.3. Diagnostic Accuracy: Classification 1

The diagnostic performance of FNA and FNB is presented in Table 3. In the FNA group 39/51 (77%) specimens were positive for malignancy compared to 29/43 (67%) in the FNB group. Of 12 samples negative for malignancy in the FNA group, ten were false negatives (14 and ten for FNB). This translates to a sensitivity of 80% versus 74%, specificity of 100% versus 100%, positive predictive value (PPV) of 100% versus 100%, negative predictive value (NPV) of 17% versus 29%, and an accuracy of 80% versus 77% for FNA and FNB, respectively (all *p* > 0.05).

### 3.4. Diagnostic Accuracy: Classification 2

Using the less stringent classification 2 (see Table 4), the sensitivity, NPV, and accuracy all increased as compared to classification 1, but there was still no statistically significant difference between FNA and FNB in any of these measures. In the FNA group 46/51 (90%) were malignant versus 38/43 (88%) in the FNB group. Of five samples negative for malignancy three were false negatives for FNA (compared to five and one for FNB). Thus sensitivity was 94% versus 97%, specificity 100% versus 100%, PPV 100% versus 100%, NPV 40% versus 80%, and accuracy 94% versus 98% for FNA and FNB, respectively.

### 3.5. Technical Outcomes

In the FNB group, 35 (81%) lesions were sampled using a 25 G needle, six (14%) lesions were sampled using 22 G, and one (2%) lesion was sampled using 19 G (needle gauge not reported in one case). Technical outcomes are reported in Table 5. Fewer needle passes were performed in the FNB group: median two (mean 2.1) compared to a median of three (mean 3.2) in the FNA group (*p* < 0.001). In the FNA group five (9.8%) patients required a repeat EUS compared to eight (18.6%) in the FNB group (*p* = 0.218). Two adverse events were reported (one gastrointestinal bleed, no blood transfusion or endoscopic therapy required, and one self-limiting episode of mild acute pancreatitis) in the FNB group compared to none in the FNA group *p* > 0.05. No technical failures were reported in either group.

In the FNA group four (7.8%) specimens were paucicellular/inadequate, three of which required repeat EUS versus three (7.0%) specimens in the FNB group of which two required repeat EUS (*p* = 1.00).

## 4. Discussion 

In this study comparing FNA to FNB, both needles demonstrate similar diagnostic performance, with FNB requiring significantly fewer needle passes to obtain sufficient diagnostic material regardless of the classification system used. To our knowledge this is the largest study comparing SharkCore FNB to conventional FNA in the diagnosis of solid pancreatic masses.

The dual classification system used in this investigation was adapted from Hewitt et al. 2012 who conducted a meta-analysis of EUS FNA in the diagnosis of pancreatic masses. Their pooled findings for sensitivity, specificity, PPV, and NPV were 85%, 98%, 99%, and 64% under classification I and 91%, 94%, 98%, and 72% under classification 2 [2]. These data are comparable to the findings presented here.

Our experience with SharkCore is similar to the findings described in a North American multicenter study using SharkCore for the diagnosis of various solid lesions. Of 250 lesions sampled, 88% were diagnostic with a median of two passes. Subgroup analysis showed that for pancreatic masses 86% were diagnostic [19]. They found a trend towards superior pathologic yield when compared to cytologic yield (87% versus 68%) but this was not statistically significant. Similarly, the Newcastle group (UK) found that SharkCore had a 90.1% sensitivity compared to ProCore™ 71.1% in a cohort of 201 patients with solid pancreatic lesions [20].

Various studies demonstrate that ProCore FNB requires fewer passes to obtain a pathologic diagnosis [13, 15–17, 19, 21]. Similarly, a recent pilot study by Adler et al. found that the SharkCore FNB required fewer passes compared to standard FNA (1.5 passes versus three passes, resp.) in a 30-patient cohort [18]. This finding supports the results of this study where a median number of two passes was needed for FNB versus three for FNA.

Of the studies investigating ProCore FNB, most did not identify a significant difference in diagnostic sensitivity between FNA and FNB for solid pancreatic masses [13–16, 19, 22]. Furthermore, when other lesions were analyzed in conjunction with pancreatic masses such as lymph nodes and gastrointestinal mass lesions, both needles still exhibited comparable levels of accuracy [17, 21, 23]. FNA sensitivity ranged within 72–92% versus 90–97.8% for FNB, and specificity ranged within 80%–100% versus 100%, respectively [13, 16, 17, 21]. This is similar to our own findings where FNA sensitivity was 80%–94% compared to 74%–97% for FNB in classification systems 1 and 2, respectively. Two studies found FNB (ProCore) to be inferior to FNA in the diagnosis of pancreatic masses [24, 25]. However both studies were small and one used a different number of passes for FNA and FNB.

Accuracy ranged within 90–94.8% for FNA and 84.6–98.3% for FNB in previously published comparative studies [15–17, 21, 24]. Not all investigations examined in this paper specified the criteria establishing malignancy; thus stringent comparisons using the two classification systems are not possible. As with most studies of EUS FNA/FNB false positives are rare and none occurred in either cohort in our study yielding specificities of 100% for both FNA and FNB under both classification systems.

Despite reports of FNB providing improved tumor type discrimination, histopathological quality, and preservation of architecture [13, 14], other studies, including our own, have not demonstrated that it increases sensitivity, although we speculate that this could be borne out if the number of needle passes was increased for FNB.

Interestingly we found that with FNA five of ten false negatives (using classification I) were neuroendocrine tumors. For FNB, seven of ten false negatives (using classification I) were pancreatic adenocarcinoma and none were eventually proven to be neuroendocrine tumors. The number of cases is too small to draw strong conclusions but this may indicate that FNB is advantageous in neuroendocrine tumors which have a spectrum of malignant potential and where a core specimen may be more important.

Although we retrospectively applied two classifications to the data, it is interesting to observe how cases were managed in reality. In the FNB group, eight patients (18.2%) required a second EUS (six pancreatic adenocarcinomas, one hemangioendothelioma, and one benign). This compares to five (9%) in the FNA group (three pancreatic adenocarcinomas and two neuroendocrine tumors). This trend is not statistically significant.

It is perhaps intuitive that, for a needle designed to obtain a core, the trade-off for obtaining more tissue comes at the expense of more adverse events. The only adverse events that occurred in the study population (a gastrointestinal bleed and an episode of pancreatitis) were in the FNB group although this was not statistically significant. To date, studies using other FNB needles have reported a similar safety profile to FNA [22] and many reported no adverse events at all [13, 14, 19, 25]. Studies with larger sample sizes are required to assess the safety profile of the SharkCore FNB.

This study was retrospective and so has inherent limitations. Other limitations are that tissue sampling was not standardized in terms of number of passes, number of needle throws, and use of suction or “slow-pull” technique. Secondly the FNA group differs significantly from the FNB group in terms of the final diagnosis; however the majority of lesions were pancreatic adenocarcinoma and neuroendocrine tumors for both groups and this is unlikely to have had a material effect on diagnostic performance. Thirdly pathological specimens were not always reported in a strict categorical format. Finally, the FNB cohort dates from when the SharkCore needle was introduced, whereas the FNA cohort represents data taken at a time when both endosonographers were very familiar with that needle. There is inevitably a learning curve with new equipment and it is possible that, with more experience and familiarity, performance could improve slightly for FNB. Lastly, the sample size reported here is relatively small.

## 5. Conclusions

In summary, the study found FNA and FNB to have comparable diagnostic accuracy and safety, with FNB requiring fewer passes. A prospective, randomized trial is warranted to establish whether FNB has an advantage over FNA.

## Figures and Tables

**Table 1:** Patient demographics.

	FNA (*n* = 51)	FNB (*n* = 43)	*p*
*Sex n (%)*			0.147
Female	29 (56.9)	18 (41.9)	
Male	22 (43.1)	25 (58.1)	
*Age*			0.756
Median (IQR)	66.0 (55.0–75.0)	66.0 (56.0–75.0)	
Mean (SD)	64.8 (12.2)	65.9 (12.7)	
Range	33.0–83.0	36.0–88.0	

FNA = fine needle aspiration; FNB = fine needle biopsy; IQR = interquartile range; SD = standard deviation.

**Table 2:** Lesion characteristics.

	FNA (*n* = 51)	FNB (*n* = 43)	*p*
*Location of lesion in pancreas, n (%)*			0.275
Head	27 (52.9)	31 (72.1)	
Uncinate	5 (9.8)	2 (4.7)	
Genu	3 (5.9)	3 (7.0)	
Body	8 (15.7)	5 (11.6)	
Tail	8 (15.7)	2 (4.7)	
*Lesion size (largest dimension, mm) *			0.787
Missing, *n* (%)	1 (2.0)	5 (11.6)	
Median (IQR)	26.0 (18.0, 34.0)	26.0 (18.0, 36.0)	
Mean (SD)	26.8 (12.8)	27.4 (11.5)	
Range	(5.0, 70.0)	(9.0, 55.0)	
*Cytologic/histologic diagnosis *			0.468
Nondiagnostic	2 (3.9)	0 (0.0)	
Benign	3 (5.9)	5 (11.6)	
Atypical	2 (3.9)	4 (9.3)	
Suspicious	5 (9.8)	5 (11.6)	
Malignant	39 (76.5)	29 (67.4)	
*Final diagnosis*			0.018
Pancreatic adenocarcinoma	37 (72.5)	31 (72.1)	
Neuroendocrine tumour	12 (23.5)	4 (9.3)	
Inflammatory	0 (0.0)	4 (9.3)	
Lymphoma	0 (0.0)	1 (2.3)	
Benign/normal	2 (3.9)	1 (2.3)	
Other	0 (0.0)	2 (4.7)	

FNA = fine needle aspiration; FNB = fine needle biopsy; IQR = interquartile range; SD = standard deviation.

**Table 3:** Diagnostic performance: classification 1.

	FNA	FNB	*p*
Sensitivity	0.80 (0.66, 0.90)	0.74 (0.58, 0.87)	0.615
Specificity	1.00 (0.09, 1.00)	1.00 (0.28, 1.00)	1.000
PPV	1.00 (0.87, 1.00)	1.00 (0.83, 1.00)	1.000
NPV	0.17 (0.02, 0.48)	0.29 (0.08, 0.58)	0.652
Accuracy	0.80 (0.67, 0.90)	0.77 (0.61, 0.88)	0.801

Values in brackets are 95% confidence interval. *p* value is based on Fisher's exact test; FNA = fine needle aspiration; FNB = fine needle biopsy.

**Table 4:** Diagnostic performance: classification 2.

	FNA	FNB	*p*
Sensitivity	0.94 (0.83, 0.99)	0.97 (0.87, 1.00)	0.626
Specificity	1.00 (0.09, 1.00)	1.00 (0.28, 1.00)	1.000
PPV	1.00 (0.89, 1.00)	1.00 (0.87, 1.00)	1.000
NPV	0.40 (0.05, 0.85)	0.80 (0.28, 0.99)	0.524
Accuracy	0.94 (0.84, 0.99)	0.98 (0.88, 1.00)	0.623

Values in brackets are 95% confidence interval. *p* value is based on Fisher's exact test; FNA = fine needle aspiration; FNB = fine needle biopsy

**Table 5:** Technical outcomes.

	FNA (*n* = 51)	FNB (*n* = 43)	*p*
*Passes*			<0.001
Unknown	1	0	
1	1 (2.0)	0 (0.0)	
2	5 (10.0)	36 (83.7)	
3	27 (54.0)	6 (14.0)	
4	16 (32.0)	1 (2.3)	
5	1 (2.0)	0 (0.0)	
*Required a repeat EUS/FNA, n (%)*			0.218
No	46 (90.2)	35 (81.4)	
Yes	5 (9.8)	8 (18.6)	
*Adverse events, n (%)*	0 (0)	2 (4.65)	0.207

FNA = fine needle aspiration; FNB = fine needle biopsy; EUS = endoscopic ultrasound.
